# Confocal Microscopy of the Cornea in Aqueous-Deficient Dry Eye Disease—A Literature Review

**DOI:** 10.3390/diagnostics14151613

**Published:** 2024-07-26

**Authors:** Radu Bucsan, Ruxandra Coroleucă, Gerhard Garhöfer, Alina Popa-Cherecheanu, Leopold Schmetterer, Raluca Iancu

**Affiliations:** 1 Department of Ophthalmology, University of Medicine and Pharmacy Carol Davila, 050747 Bucharest, Romania; radu-gheorghe.bucsan@drd.umfcd.ro (R.B.); ruxandra.coroleuca@umfcd.ro (R.C.); raluca.iancu@umfcd.ro (R.I.); 2 Department of Ophthalmology, Emergency University Hospital Bucharest, 050098 Bucharest, Romania; 3 Department of Clinical Pharmacology, Medical University of Vienna, 1090 Vienna, Austria; gerhard.garhoefer@meduniwien.ac.at; 4 Singapore Eye Research Institute, Singapore National Eye Centre, Singapore 168751, Singapore; 5 Ophthalmology and Visual Sciences Academic Clinical Program, Duke-NUS Medical School, National University of Singapore, Singapore 169857, Singapore; 6 SERI-NTU Advanced Ocular Engineering (STANCE), Singapore 639798, Singapore; 7 School of Chemical and Biological Engineering, Nanyang Technological University, Singapore 637371, Singapore; 8 Center for Medical Physics and Biomedical Engineering, Medical University Vienna, 1090 Vienna, Austria; 9 IOB—Institute of Molecular and Clinical Ophthalmology, 4031 Basel, Switzerland; 10 Fondation Ophtalmologique Adolphe De Rothschild, 75019 Paris, France

**Keywords:** confocal microscopy, cornea, dry eye, Sjogren syndrome

## Abstract

Background: In vivo confocal microscopy (IVCM) is a vital tool in studying dry eye disease (DED), providing insights into morphological changes at ocular surface unit levels. This review presents the main differences in corneal structure between aqueous-deficient dry eye disease (AD-DED) and normal eyes. Methods: A comprehensive search of PubMed, Web of Science, Embase, and MEDLINE databases from January 2000 to December 2023 was conducted. The study selection process, as well as data selection and examination, were independently performed by two members of the review team. Results: The review reveals a consistent decrease in corneal surface epithelial cell density in AD-DED cases compared to a control group, but conflicting data on basal epithelial cell density. Notably, the abnormal hyperreflectivity of keratocytes in patients with Sjogren’s syndrome was recorded, and there was a significant keratocyte density in AD-DED subjects compared to evaporative DED and control groups. Studies also found a decrease in sub-basal nerve density, increased tortuosity, and the fragmentation of nerve fibers. Dendritic cell density and dendritic cell dendrites increase in AD-DED patients compared to healthy subjects. Conclusions: IVCM is a powerful tool for enhancing our understanding of the pathophysiological mechanisms underlying DED. However, the review underscores the urgent need to standardize the terminology, analysis, and units used for accurate interpretation, a crucial step in advancing our knowledge of DED.

## 1. Introduction

In 2007, The International Dry Eye Workshop (DEWS) [1] aimed to standardize the definition and classification of dry eye. Dry eye is “a multifactorial tear and ocular surface disease that results in discomfort, visual disturbance, and tear film instability with potential damage to the ocular surface”. DEWS recognizes the complexity of dry eye and emphasizes the importance of symptoms and objective signs in its diagnosis [2]. Ten years later, The 2017 International Dry Eye Workshop II (DEWS II) [3,4] redefines dry eye as: “a multifactorial disease of the ocular surface, characterized by a loss of tear film homeostasis and accompanied by ocular symptoms, in which tear film instability and hyperosmolarity, inflammation and damage to the ocular surface, as well as neurosensory abnormalities, play etiological roles”.

Dry eye disease (DED) global prevalence is estimated at approximately 12% [5], so DED remains one of the common public health concerns. From a clinical point of view, DED has two subtypes: aqueous-deficient DED (AD-DED), defined by decreased tear secretion, and hyper-evaporative DED, characterized by increased tear evaporation, although many patients have both subtypes present [3]. Approximately 10% of the patients with DED have isolated aqueous-deficient disorder [6]. DED represents a social and economic burden due to the reduced quality of life, loss of professional productivity, and psychological affliction due to ocular surface pain and visual disturbance [2,3,4].

In vivo confocal microscopy (IVCM) is a valuable and minimally invasive imaging technique that allows the visualization and assessment of various structures on the ocular surface at the cellular level. It provides real-time, high-resolution images that can help evaluate the different components of the ocular surface [7]. IVCM can provide high-quality images of epithelial cells, keratocyte morphology, sub-basal nerve cells, dendritic cells, and endothelial cells. By providing detailed and real-time cellular imaging, IVCM aids in diagnosing, monitoring, and researching various ocular surface conditions [8]. It helps understand the underlying pathophysiology, assess response to treatment, and guide therapeutic interventions for patients with DED [9].

Studies on IVCM have provided valuable insights into the changes in corneal epithelium and stroma in DED [10]. These changes indicate underlying inflammatory processes and metabolic changes associated with the condition [11].

These IVCM findings provide objective evidence of the structural changes in the cornea of DED patients. The presence of abnormal keratocytes and altered cell densities in the epithelium highlights the impact of inflammation and metabolic dysregulation on the pathogenesis of DED [12].

By identifying these cellular changes, IVCM not only aids in diagnosing and monitoring DED but also provides valuable insight into the underlying mechanisms. The capability to determine the distinct causes of an individual patient’s symptoms may lead to proper treatment. This knowledge may contribute to developing targeted therapies aimed at reducing inflammation, restoring normal cell function, and improving the ocular surface health in patients with dry eye [2,3,4,13].

Our goal for this article is to review the literature on the clinical use of IVCM in DED, focusing on the studies published in the last ten years. We will summarize the main findings of IVCM in AD-DED assessment and its limitations.

## 2. Methods

For the literature review, we searched the PubMed, Web of Science, Embase, and MEDLINE databases from January 2000 to December 2023 using the following keywords: confocal microscopy, dry eye, Sjogren syndrome, and cornea. This review is not intended to be a systematic or exhaustive literature review but a narrative summary of the results of the in vivo confocal microscopy analysis of patients with DED due to AD-DED. The results included the histopathological changes found with IVCM in tissues affected by aqueous-deficient DED, namely the cornea. In analyzing the specialized literature, we included articles selected according to the above criteria, and some of their reference articles cited. The initial database search with the keywords identified 104 articles. Relevant original and review articles reference list was screened to identify further eligible studies related to corneal structure evaluation by IVCM, leaving 46 results. After full-text retrieval, 23 studies were included.

## 3. Results

### 3.1. Corneal Epithelium

Several studies were selected to describe the changes in the corneal structures of patients with AD-DED (Table 1).

Kasikci et al. [13] found a significant decrease in corneal superficial epithelial cell density (738 ± 289 cells/mm^2^ for the aqueous type and 1550 ± 445 cells/mm^2^ for the control group) and a significantly increased density of corneal basal epithelial cell in the aqueous type compared to the control group. Benitez del Castillo et al. [18,19] compared four groups that described a significant decrease in corneal surface epithelial cell density in cases with AD-DED in comparison with a control group patients: primary Sjogren’s syndrome (SS), non-Sjogren’s syndrome (NSS), control group over 60 years of age (NL ≥ 60), and control group under 60 years of age (NL < 60), and found a statistically significant decrease in the density of superficial epithelial cells in the case of SS (741 ± 306 cells/mm^2^) and NSS (1022 ± 331 cells/mm^2^) patients compared to NL ≥ 60 (1523 ± 294 cells/mm^2^) and NL < 60 (1529 ± 341 cells/mm^2^). However, no significant difference was found among the four groups regarding basal epithelial cell density: SS (5746–6599 cells/mm^2^), NSS (5425–6044 cells/mm^2^), NL ≥ 60 (5168–6062 cells/mm^2^), and NL < 60 (5217–6348 cells/mm^2^) [15]. Villani and collaborators [20], in a study comparing a group of patients with Sjogren’s syndrome with a control group, confirmed a statistically significant decrease in the density of superficial epithelial cells (985.05 ± 107.57 cells/mm^2^) compared to the control group (1485 ± 133.74 cells/mm^2^), as well as a decrease in corneal thickness (514.75 ± 17.14 μm versus 559.23/−24.46 μm); however, unlike Benitez del Castillo et al., a statistically significant increase in the density of basal epithelial cells (6197.37 ± 180.34 cells/mm^2^ vs. 5861.65 ± 260.40 cells/mm^2^) was observed. Older studies, like Tuominen et al. [21], did not observe a difference in basal epithelial cell density between patients with Sjogren’s syndrome (SS) and those in the control group. Still, there was a decrease in corneal thickness in the former group (515.9 ± 22.0 μm vs. 547.4 ± 42.0 μm), as well as several morphological changes, including the irregularities of superficial epithelial cells, hyperreflectivity of anterior keratocytes, and decreased epithelial thickness.

Erdélyi et al. [22] conducted another study with four groups: patients with aqueous dry eye syndrome (S), patients with thyroid ophthalmopathy (D), patients with chronic lagophthalmos (L), and a control group (N). Their results found a decreased density of central cornea superficial epithelial cells in all three groups of patients (S = 843 ± 198 cells/mm^2^, D = 920 ± 89 cells/mm^2^, and L = 1.002 ± 196 cells/mm^2^) compared to the control group (1.212 ± 242 cells/mm^2^) with a significant difference (*p* < 0.01) between S and N groups. They also recorded a non-significant (*p* = 0.159) decrease in central cornea basal epithelial cell density in the S group (9.131 ± 1.701 cells/mm^2^) compared to the control group (9.858 ± 99 cells/mm^2^), as well as a significant decrease (*p* < 0.01) in central corneal thickness for S group (523 ± 25 μm) compared to the control group (544 ± 25 μm) and for L group (476 ± 45 μm) compared to D group (544 ± 37 μm). In addition, Erdélyi found the hyperreflectivity of the peripheral stroma in patients with aqueous dry eye syndrome (A). Zhang X et al. [23] compared three groups of patients: moderate dry eye (MDE), moderate–severe dry eye (MSDE), and a control group (NL). Their results confirmed a statistically significant decrease in the density of superficial epithelial cells in the MDE (890 ± 197 cells/mm^2^) and MSDE (746 ± 125 cells/mm^2^) groups compared to the control group (1228 ± 248 cells/mm^2^), but contrary to some previously presented results, they also found a statistically significant decrease in basal epithelial cell density in the two groups MDE (9234 ± 1365 cells/mm^2^) and MSDE (8634 ± 998 cells/mm^2^) compared to the control group (11,307 ± 1876 cells/mm^2^).

Lee et al., in a recent study [16], also demonstrated morphological changes in the corneal epithelium in DED subjects. The density of superficial epithelial cells decreased in non-Sjögren DED and Sjögren DED compared with controls.

In an even more recent study by Matsumoto et al. [17], the IVCM evaluation of DED subjects with Sjögren syndrome showed that the corneal superficial epithelial cell density was significantly lower in Sjögren syndrome subjects than in control subjects.

### 3.2. Corneal Stroma

**Figure 1:**
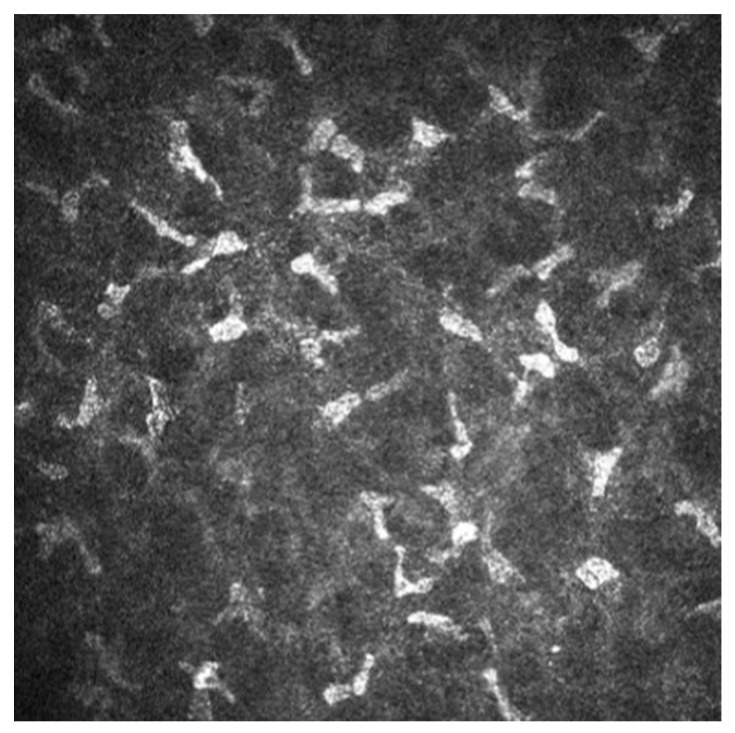
Abnormal hyperreflectivity of keratocytes in a patient with Sjogren’s syndrome.

Tuominen [21] highlighted the abnormal hyperreflectivity of keratocytes in patients with Sjogren’s syndrome (SS), which may present morphological evidence of activation (as seen in Figure 1).

The optical density of keratocytes in the anterior stroma was higher than in the control group, which also showed vacuoles [21]. In the four groups compared by Benitez del Castillo et al. [18], anterior stromal cell density was higher in the group with primary Sjogren’s syndrome (SS = 1200–1497 cells/mm^2^) than in the other groups: non-Sjogren’s syndrome (NSS = 988–1379 cells/mm^2^), control group over 60 years of age (NL ≥ 60 = 931–1219 cells/mm^2^), and control group under 60 years of age (NL < 60 = 965–1248 cells/mm^2^). At the same time, the posterior stromal cell density was higher in the SS group (729–887 cells/mm^2^) than in the NSS group (687–903 cells/mm^2^) and NL < 60 group (645–837 cells/mm^2^) than in the NL ≥ 60 group (682–854 cells/mm^2^). None of these differences was statistically significant. Kasikci et al. (see Table 1) also demonstrated a considerable keratocyte density in AD-DED subjects compared to evaporative DED and control groups [13].

### 3.3. Corneal Nerve Density and Morphology

**Figure 2:**
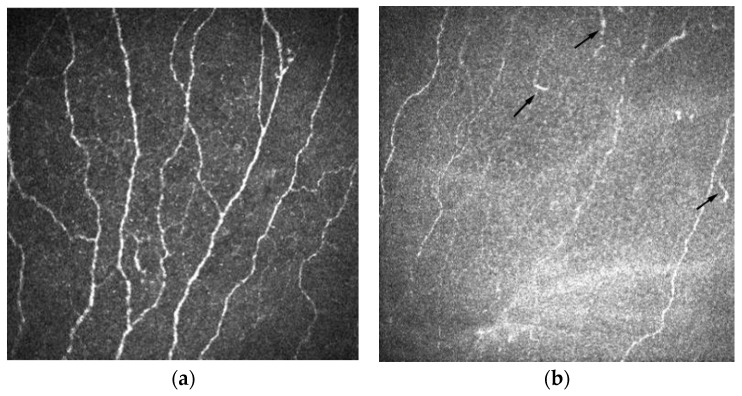
(**a**) Normal image of sub-basal nerve plexus, image property of Heidelberg Engineering GmbH. Sub-basal nerve plexus without dendritic cells in a healthy eye. (**b**) Corneal confocal image highlighting: decreased nerve density, dendritic cells at the level of sub-basal nerve plexus with dendritic processes (arrows) in a patient with dry eye.

Several studies were selected to describe the changes in the corneal nerve density and morphology in AD-DED subjects (see Table 1, Figure 2a,b) compared with control subjects. These studies were conducted on subjects with DED related to Sjogren’s syndrome [13,14,17].

Kasicki et al. [13] found a significant increase in nerve pilling and nerve folds in the AD-DED and evaporative dry eye (EDE) groups compared to healthy controls. Still, no significant changes in neural reflectivity were observed among the three groups.

Cox et al. [14] determined statistically significant differences in all the parameters (total nerve density, main nerve density, branch nerve density, total nerves/frame, main nerves/frame, and branch nerves/frame) evaluated for corneal nerves between control and AD-DED. Patients with AD-DED demonstrated compromised corneal subbasal nerves compared to the control group, respectively, with decreased values in AD-DED for all the analyzed corneal nerve parameters.

In their paper, Matsumoto et al. [17] compared a group of Sjögren’s syndrome (SS) patients to a control group and found an increase in nerve tortuosity and beading. Still, the sub-basal nerve reflectivity grade showed no significant difference between the two groups.

Hoşal et al. compared a group of patients with primary Sjogren’s syndrome (SS), a group of patients with non-Sjogren’s dry eye syndrome (NSS), and a control group (NL) and found no difference in terms of sub-basal nerve density or stromal density. They only recorded an increase in the diameter of the stromal nerves in patients with SS, but this was not statistically significant (*p* > 0.05) [24].

Contrary to Hoșal’s results, Zhang M et al., comparing three similar groups (SSI, NSS, and NL), found an increase in sub-basal density both in SSI patients (1745.4 ± 414.7) and in NSS patients (1423.5 ± 609.5) compared to the control group (1315.7 ± 664.7). For the SSI patients, the difference was statistically significant. Their results were reported as the total nerve fiber length (μm)/frame; however, the frame size was not mentioned. Morphologically, increased tortuosity, fragmentation of nerve fibers, and branching were found [25].

Benitez del Castillo et al. compared four groups of patients: primary Sjogren’s syndrome (SSI), non-Sjogren’s syndrome (NSS), control group aged over 60 years (NL ≥ 60), and control group aged under 60 years (NL < 60), and demonstrated a statistically significant decrease in the sub-basal nerve density (total length of nerve fibers in μm/an area of 74,340 μm^2^) in the SSI (511 ± 106) and NSS (591 ± 90) group, vs. NL < 60 (787 ± 105) and NL ≥ 60 (620 ± 92) in the control groups. They also found increased tortuosity and fragmentation of nerve fibers [19].

Villani et al. recorded a statistically significant decrease in the sub-basal density in patients with Sjogren’s syndrome (3.34 ± 0.76) compared to the control group (5.10 ± 0.79). Density was measured as the number of nerves/frames without reference to frame size. They also found an increase in tortuosity and reflectivity [20].

Tuisku et al. published a comparative study between patients with primary Sjogren’s syndrome (SSI) and a control group (NS). They found an increase in the diameter of the stromal nerves in the SSI group. They found no difference in the sub-basal density (number of nerves/0.136 mm^2^) between the SSI group (5.9 ± 2.2) and the control group (6.1 ± 2.5) [26].

Zhang X et al. compared a group of patients with moderate dry eye (MDE) with a group of patients with moderate to severe dry eye (MSDE) and a control group (NL) and found an increase in the nerve fiber tortuosity in both groups of patients with dry eye [23].

In another study, Cruzat et al. compared three groups of patients with dry eye associated with three types of keratitis (bacterial—BK, fungal—FK, and Acanthamoeba—AK) with a control group. They found a statistically significant decrease in the sub-basal density (reported as nerve length in μm/0.16 mm^2^ frame) in all three types of keratitis BK (824.0 ± 1050.7), FK (956.9 ± 1093.0), and AK (215.6 ± 575.4) compared to the control group (3913.9 ± 507.4) [27].

### 3.4. Inflammatory Cells

In AD-DED subjects, multiple authors noticed modified parameters of inflammatory cells.

Kasikci et al. [13] found significantly increased dendritic cell number, area, density, and size, *p* < 0.01 for all the parameters (see Table 1).

Matsumoto et al. [17] revealed that the mean inflammatory cell density at the subbasal epithelial area was significantly higher in AD-DED patients than in healthy control subjects (87.0 ± 52.5 cells/mm^2^ and 17.3 ± 18.8 cells/mm^2^, respectively).

Lin et al. [28] showed increased dendritic cell density in the central cornea of primary Sjogren’s syndrome patients. They also showed a statistically significant increase in the peripheral dendritic cell density in patients with primary Sjogren’s syndrome (SS = 157.2 ± 29.7 cells/mm^2^) and those with non-Sjogren’s dry eye syndrome (NSS = 106.9 ± 10.5 cells/mm^2^) compared to the control group (NL = 90.7 ± 8.2 cells/mm^2^) as well as a statistically significant increase in the peripheral non-dendritic leukocytes in SS patients (84.2 ± 36.8 cells/mm^2^) compared to NSS patients (8.4 ± 3.1 cells/mm^2^) and the NL control group (4.3 ± 1.3 cells/mm^2^). The same results were obtained in a more recent study conducted by Aggarwal et al. in 2021: for DED patients, they found a statistically significant increase in dendritic cell density compared to controls (93.4 ± 6.3 vs. 25.9 ± 3.9 cells/mm^2^; *p* < 0.001) (see Table 1) [15]. Regarding the density of inflammatory cells at the central level, Lin et al. showed a statistically significant increase in dendritic cells in SSI (127.9 ± 23.7 cells/mm^2^) and NSS (89.8 ± 10.8 cells/mm^2^) patients compared to the control group NL (34.9 ± 5.7 cells/mm^2^), a statistically significant increase in dendritic cell dendrites in SSI (46.1 ± 17.3 cells/mm^2^) and NSS (9.9 ± 1.7 cells/mm^2^) patients compared to the NL control group (3.2 ± 0.5 cells/mm2), and a statistically significant increase in non-dendritic leukocytes in the SSI (49 ± 12.9 cells/mm^2^) and NSS (4.6 ± 1.0 cells/mm^2^) patient groups compared to the NL control group (1.6 ± 0.6 cells/mm^2^). The morphology of dendritic cells has also been modified in DED subjects. As Aggarwal et al. demonstrated, the dendrite number and the size of the cells are significantly larger in DED subjects (3.3 ± 0.1, 106.9 ± 4.7 μm^2^, 403.8 ± 20.1 μm^2^) than in controls (2.3 ± 0.1, 62.5 ± 5.7 μm^2^, 241.4 ± 24.4 μm^2^, *p* < 0.001) [15].

## 4. Discussion

IVCM is a valuable tool for improving our understanding of the pathophysiological mechanisms underlying DED. Recent IVCM studies have demonstrated its potential for various clinical applications in treating DED—the present study aimed to summarize the main differences in the corneal structure between AD-DED and normal eyes.

Although a series of IVCM studies have demonstrated a decrease in superficial cell density and an increase in basal cell density in the corneal epithelium of patients with DED [13,20,22,29], del Castillo found no difference in basal cell density between DED patients and controls [18] and Zhang X et al. reported a decrease also in basal cell density [23]. This suggests an interruption in the normal cellular regeneration process, leading to epithelial abnormalities. A decreased superficial cell density may impair barrier function and increase susceptibility to epithelial damage [30,31]. Most studies showed a significantly higher basal epithelial cell density in AD-DED compared to control groups; when this parameter was compared between the two types of dry eye (aqueous-deficient and evaporative), the difference in basal cell density was not significant [13,18,32]. However, the inconsistencies related to basal cell density changes require further investigation.

In addition to epithelial changes, IVCM revealed changes in the corneal stroma of patients with DED. A common finding is the presence of abnormal hyperreflective keratocytes [13,20,33,34]. These hyperreflective keratocytes are often referred to as ‘activated’ cells and are thought to be in a state of metabolic activation induced by pro-inflammatory mediators [19,20,33]. This activation is thought to respond to the inflammatory environment in DED patients.

Tuominen et al. concluded that in patients with SS, the thickness of the central cornea and stroma is low due to metabolic changes and inflammatory processes [21].

The cornea is one of the most innervated tissues of the body, with a significantly higher density of nerve endings than the skin (200–300 times higher) [35]. These nerves in the cornea perform sensory functions and are known as nociceptors. Nociceptors play a crucial role in maintaining the overall health and function of the cornea [36].

One of the main functions of corneal nerves is to maintain homeostasis. They regulate tear secretion and distribution, which are essential for maintaining the stability and lubrication of the ocular surface. Corneal nerves detect changes in the composition of the tear film, and reflex triggers tear production or changes in tear composition to ensure that the corneal tissue remains moist and protected [36].

The corneal nerves also play a vital role in the wound-healing process. When the cornea is damaged, nociceptors detect the damage and send signals to initiate healing. These signals trigger various cellular responses, including the migration and proliferation of epithelial cells, release of growth factors, and recruitment of inflammatory cells to promote the repair of damaged tissue [37,38]. In addition, corneal nerves are responsible for sensing the environment and protecting the cornea from potential damage. They detect and transmit signals responding to various stimuli, such as mechanical forces, temperature changes, and chemical irritants. This sensory feedback helps maintain the cornea’s integrity by triggering protective reflexes, such as blinking or lacrimation, to eliminate or avoid potentially harmful substances or conditions [38]. In summary, the dense network of corneal nerves, especially nociceptors, performs essential functions in maintaining corneal homeostasis, contributing to the wound-healing processes, and protecting the cornea from potential damage. The complex interactions between the corneal nerves and the ocular surface are crucial to the overall health and function of the eye.

IVCM has emerged as a valuable tool for studying corneal nerves, particularly the sub-basal nerve plexus. It allows a detailed examination of nerve morphology, density, and changes induced by various diseases or surgical procedures [39,40]. One of the advantages of IVCM is its ability to provide high-resolution images of corneal nerves, particularly in the central cornea near the apex. Using tangential imaging techniques, IVCM can capture clear and detailed images of the sub-basal nerve plexus. This region is of particular interest because it contains a dense network of nerves that plays a crucial role in the sensory function and health of the cornea [41,42].

However, it is essential to note that IVCM has some limitations in imaging specific corneal nerve structures. It lacks sufficient resolution to visualize nerve endings in the epithelium, which are fine branches extending into the cornea’s superficial layers. In addition, IVCM may not adequately capture the very-small-diameter nerves in the sub-basal plexus [41].

Another challenge associated with IVCM is the limited imaging range of the devices. For example, an HRT laser scanning confocal microscope (Heidelberg Engineering GmbH, Heidelberg, Germany), commonly used in studies, typically provides images of an area of 400 μm × 400 μm. This can make it laborious to specifically locate and consistently image the same cornea region, essential for longitudinal studies and disease progression monitoring.

Despite these limitations, IVCM remains a valuable tool for studying corneal nerves and their changes under various conditions. It provides insights into the structural changes and density of the corneal nerves, which is valuable information for understanding eye disease pathophysiology and evaluating treatment responses. IVCM was used to evaluate corneal nerve changes in non-SS- or SS-associated AD-DED patients. These studies demonstrated changes in nerve density, tortuosity, and fragmented (bead-like) formations, providing insights into the neuropathic mechanisms underlying dry eye conditions [26,43].

Several studies have described findings related to the density, morphology, and regeneration of nerves in the sub-basal plexus of the cornea in patients with aqueous lacrimal deficiency (ATD) and Sjogren’s syndrome (SS).

- Decreased nerve density: Benítez del Castillo et al. found that non-SS and SS aqueous tear deficiency (ATD) groups had decreased nerve density in the sub-basal plexus compared to normal subjects under 60. This suggests a reduced corneal innervation in individuals with ATD [18].
- Beading nerves: ATD groups showed more beading nerves in the sub-basal plexus than control subjects in different age groups. Beaded nerves are characterized by swelling or varicosities along nerve fibers and are signs of nerve damage or degeneration.
- Nerve tortuosity: Villani et al. reported a decreased number and increased tortuosity of sub-basal nerves in patients with ATD compared to controls. Increased sinuosity indicates the abnormal coiling or twisting of nerve fibers, which may reflect nerve degeneration or structural changes [20].
- Neoinnervation: Tuominen et al. observed neoinnervation in the superficial nerve plexus (SNP) in 40% of the eyes with SS. This refers to forming new nerve branches or extensions that may represent attempts at nerve regeneration or repair [21].

The authors hypothesize that these outcomes may indicate regeneration of sensory nerves, as similar observations were made in animal studies using capsaicin (a neuropeptide-releasing agent) [44] and overexpression of nerve growth factor (NGF). Capsaicin treatment in the cornea of newborn rats induced nerve sprouting, while NGF overexpression resulted in hypertrophy of the peripheral nervous system [44].

These findings suggest that individuals with ATD and SS may exhibit corneal nerve density, morphology, and regeneration changes. These changes likely contribute to the ocular surface symptoms and complications observed in these conditions, such as dryness, discomfort, and corneal damage.

A study by Tuisku et al. [26] revealed some distinct findings regarding corneal sub-basal nerves and dendritic cells (DCs) in patients with Sjogren’s syndrome (SS) compared to control subjects.

- Sub-basal corneal nerve density: Unlike the previously mentioned study, Tuisku et al. [26] found that sub-basal corneal nerve density was similar between SS and control subjects. This suggests that the total number of nerves in the sub-basal plexus may not be significantly affected by the SS.
- Thickening and “coning” of structures: The study did see thickening and cone-like structures in the corneal nerves of SS patients. These structural changes indicate an abnormal morphology and suggest potential nerve damage or degeneration.
- Symptoms of hyperalgesia and irritation: The presence of thickened and cone-type nerves in the SS cornea is accompanied by hyperalgesia, which refers to an increased sensitivity to pain or discomfort. This was assessed by using an air jet esthesiometer. These findings suggest that altered nerve morphology may contribute to the increased sensitivity and perception of irritation symptoms in patients with SS.
- Increased density of dendritic cells: This study also noted an increased density of dendritic cells around the corneal nerves in the SS. Dendritic cells are immune cells involved in recognizing and responding to foreign substances. Dendritic cell density correlates with the severity of irritation symptoms, suggesting a potential role in SS’s inflammatory response and symptomatology [26].

This study indicates that while sub-basal corneal nerve density may not be significantly affected in SS, distinct structural abnormalities and increased dendritic cell density are associated with altered nerve morphology. These outcomes provide insights into the potential mechanisms underlying sensory abnormalities and inflammatory responses observed in patients with SS.

Confocal microscopy studies have provided evidence supporting the involvement of the sub-basal nerve plexus in DED. They show increased nerve tortuosity in patients with DED [19,20,32], indicating structural changes in the nerve fibers. Additionally, an increased density of fragmented (bead-like) formations [20,32] was observed within the nerves.

The interpretation of these changes in sub-basal nerve tortuosity and beading remains a matter of debate and requires further investigation. Some studies have suggested that these changes may indicate nerve damage, reflecting neurodegenerative aspects of DED. However, an alternative interpretation is that these changes may reflect increased metabolic activity of nerve fibers in response to abnormal epithelial changes and neuroinflammation.

It is important to note that there is conflicting data regarding these changes and their correlation with the sensitivity. Some studies have reported a correlation between nerve tortuosity and decreased sensitivity, suggesting that nerve changes contribute to the development of DED symptoms. Other studies did not find a significant correlation between nerve changes and symptoms [19,20,21,24,25,43,45].

Further research is required to understand better the significance of sub-basal nerve tortuosity and beading in DED. This includes investigating their relationship with symptoms, exploring the mechanisms underlying these changes, and determining their potential as diagnostic or prognostic markers.

IVCM studies have shown that the density of epithelial dendritic cells, considered antigen-presenting cells and other inflammatory cells, is increased in the central and peripheral corneas of DED patients [27,28]. This indicated the presence of an inflammatory response on the ocular surface. Aggarwal et al., in 2021, concluded that there is a correlation between dendritic cell density and morphology with the clinical severity of DED; while dendritic cell density is increased in DED, morphological changes are seen only in severe cases [15].

A dynamic assessment of inflammatory cell density in the central cornea using IVCM may be valuable for assessing the severity of DED. It provides quantitative information about the level of inflammation, which can help determine the appropriate clinical treatment for each patient. Furthermore, this technique may help evaluate the efficacy of anti-inflammatory drugs in clinical trials by monitoring changes in inflammatory cell density over time [7,29].

By using IVCM to visualize and quantify inflammatory cell density, clinicians and researchers can gain insights into the underlying pathophysiology of DED and track the effectiveness of interventions to reduce ocular surface inflammation. In addition to DED, multiple corneal pathologies, such as keratomycosis, keratoconus, corneal grafting, and refractive surgery, can benefit clinically from IVCM.

However, IVCM imaging of the human cornea has several limitations. One of the significant problems with this technique is that, in follow-up studies, it is challenging to find the same lateral position again because of a lack of sufficient landmarks. IVCM imaging requires physical contact with the corneal epithelium, which can cause patient discomfort or pain [46]. IVCM imaging has a limited field of view (typically ~400 µm × 400 µm); therefore, imaging larger areas of the cornea requires multiple images ‘puzzling’, thus significantly increasing the image acquisition and processing time [46]. Although optical coherence tomography (OCT) imaging is very promising for corneal cross-sectional imaging, IVCM still offers superior lateral resolution and higher imaging quality [47]. The most used methodology in clinical research settings involves randomly selecting a set number of images with sufficient quality [48]. The sampling strategy of adequate images has been shown to produce sufficient accuracy. However, for longitudinal or clinical monitoring purposes, real-time widefield imaging capabilities may provide more insight into regional changes in the cornea over time or following exposure to various stimuli [49,50]. OCT is an optical imaging technique that generates cross-sectional and volumetric images of biological tissues with cellular-level resolution. Recent developments in broadband light sources and high-speed, large pixel number cameras have resulted in the development of spectral domain and full-field ultrahigh resolution OCT (UHR-OCT) technology with axial resolution close to or below one μm, which is suitable for imaging the cellular and subcellular structure of biological tissue [46,51]. UHR-OCT is easier to use, but the required resolution has not yet been achieved. These advancements may improve the applicability of corneal confocal microscopy and improve the efficiency of observing corneal microstructures to understand their biological significance [52].

## 5. Conclusions

IVCM is a valuable tool for improving our understanding of the morphological changes and pathophysiological mechanisms underlying DED. It allows us to evaluate the ocular surface at the cellular level.

Confocal microscopy serves as a bridge between the traditional ex vivo histology and in vivo investigations of ocular pathology, thereby contributing to both clinical research and ophthalmological practice. Nevertheless, image interpretation requires excellent experience, and their analysis must be performed in a clinical context.

## Figures and Tables

**Table 1:** Selected study findings, focusing on the studies published in the last ten years, related to statistically significant changes in the corneal structure in DED compared to a control group (*p*-values are listed accordingly).

Study	Corneal Epithelium		Corneal Stroma	Corneal Nerves		Dendritic Cells Density
	Superficial Epithelial Cell Density	Basal Epithelial Cell Density	Keratocyte Density	Nerves Density	Nerves Morphology Changes	
Kasikci 2023 [13]	Corneal superficial epithelial cell density significantly decreases in DED cases	Corneal basal epithelial cell density significantly increased in AD-DED	Significantly increased in the AD-DED	The number and density of sub-basal nerves significantly decrease in AD-DED	An increase was found in neural pilling and folding	Significant increase in dendritic cell density, size, number, and area in AD-DED
Cox 2021 [14]				Statistically significant lower total (*p* = 0.026) and main (*p* < 0.001) nerve density in DED	Statistically significant lower total (*p* = 0.001) and main (*p* = 0.001) and branch (*p* = 0.004) nerve numbers in DED	
Aggarwal 2021 [15]						Statistically significant increase in dendritic cell density in DED (*p* < 0.001)
Lee 2018 [16]	Statistically significant decrease in both the non-Sjögren DED (*p* < 0.05) and Sjögren’s syndrome DED groups (*p* < 0.01)	Statistically significant decrease in both the non-Sjögren DED (*p* < 0.01) and Sjögren’s syndrome DED groups (*p* = 0.01)				
Matsumoto 2020 [17]	Statistically significantly lower in AD-DED compared with control subjects (*p* < 0.0001) *			Statistically significant lower in AD-DED than control subjects (*p* < 0.0001).	Morphological abnormality of nerve fibers was observed in AD-DED patients	Statistically significantly higher in AD-DED patients compared with control subjects (*p* < 0.0001)

* the corneal superficial epithelial cell area was significantly larger in AD-DED subjects than in control subjects (*p* = 0.007).

## Data Availability

No new data were created or analyzed in this study. Data sharing is not applicable to this article.
